# Motivational Drivers behind the Consumption of Dietary Supplements by Leisure-Time Athletes

**DOI:** 10.3390/foods12163044

**Published:** 2023-08-14

**Authors:** Ildikó Kovács, Fanny Liska, Zoltán Veres

**Affiliations:** 1 Department of Marketing, Faculty of International Management and Business, Budapest Business School, University of Applied Sciences, Markó utca 29-31, 1055 Budapest, Hungary; kovacs.ildiko@uni-bge.hu; 2 Department of Marketing, Faculty of Business and Economics, University of Pannonia, Egyetem utca 10, 8200 Veszprém, Hungary; liska.fanny@gtk.uni-pannon.hu

**Keywords:** leisure-time athletes, dietary supplement consumption, consumer motivational drivers, supplementation prevalence, self-reported motivation

## Abstract

The prevalence of dietary supplement use has been growing continuously worldwide. Nevertheless, limited research evidence is available on dietary supplement usage related to the segment of leisure-time athletes with the determinants of motivational drivers, sport types, and demographic characteristics. This research, which was conducted in Hungary among leisure-time athletes, aims to learn about the demographic and sports characteristics of consumers who identified themselves as active dietary supplement consumers in the survey. The motivational drivers for consuming dietary supplements and their differences, as well as the frequency of dietary supplement consumption in each sports category, are examined. The findings of the study provide valuable insights into the key motivational drivers, among which health preservation, improvement in physical well-being, and cartilage protection are predominant. The results show that there are significant differences between segments taking part in different leisure-time sport activities and age groups when it comes to dietary supplement usage. Three different segments were identified based on the motivational drivers examined. As a limitation, to note is that due to the sample size, the study can be considered as prospective. In practical terms, the results of the study can be used to support marketing projects that aim to reach leisure-time sport athletes.

## 1. Introduction

The United States 103rd Congress defined the term “dietary supplement” (DS) in the Dietary Supplement Health and Education Act (DSHEA) of 1994 [1]. A dietary supplement is a product taken by mouth that contains a “dietary ingredient” intended to supplement the diet. The “dietary ingredients” in these products may include vitamins, minerals, herbs or other botanicals, amino acids, and substances such as enzymes, organ tissues, glandulars, and metabolites. The use of DSs has been steadily increasing all over the world in recent decades, and approximately 50–75% of the population have taken them routinely, while almost half have done so regularly [2,3]. The global dietary supplement market was valued at USD 149.50 billion in 2021 and is expected to grow at an 8.50% CAGR to USD 240.90 billion by 2028, according to Facts and Factors [4]. Research has shown that active users mostly comprise consumer groups whose mineral intake is higher than those who do not consume minerals [5]. Similar results were obtained for vitamin products [6]. Moreover, the use of protein supplements is becoming an increasingly accepted lifestyle practice among amateur athletes. Many amateur users believe that protein supplements are essential to achieve their sporting goals, and they tend to make mistakes by incorporating an inappropriate (typically too large) amount of protein into their diets [7].

Sales figures show that the consumption of sports supplements continues to grow among young athletes, and research shows that young athletes have become the main targets for marketing [8]. This study aims to gain insight into the motives determining dietary supplement use by leisure-time athletes. The latter means a person who engages in sports during his/her free time but does not compete in a particular sport [9]. To this end, dietary supplementation and the motivational determinants in four different sports categories were investigated.

### 1.1. Consumption Motivations

Previous research on the consumption of food supplements and consumer motivations, as can be seen below, has revealed that there are different motivations behind the widespread use of food supplements in both leisure and competitive sports. For athletes, research shows that dietary supplements are intended to be used primarily for the preservation of health, and as a contribution towards balanced nutrition and physical well-being. The preservation of health refers herein to the conscious and proactive efforts individuals undertake to maintain or improve their physical, mental, and emotional well-being. Conner et al. [10] found evidence that individuals who place a high value on their own health are more inclined to take dietary supplements by way of precaution. To maximize performance, athletes often turn to supplements with the belief that they will help them stay competitive and healthy [11], and boost the immune system, recovery, and overall athletic performance [12]. According to research among Swiss adults, four different perceptions of utility that consumers associate with the consumption of dietary supplements can be identified: nutrient balance, fitness as a trend, health and well-being, and muscle modulation and competitive performance [13]. Research by Aragon et al. [14] examined the most often used diet categories. Their research found that diets typically focus on reducing body weight or increasing muscle mass.

Examining socio-cognitive and psychosocial factors among dietary supplement users, Pajor et al. [15] found that individuals’ consumption of dietary supplements coincides with the following motivational factors: susceptibility to promotions, seeing examples of supplement use in their environment, feeling like they have higher chances of becoming sick, and positive attitudes towards supplements. In America, a survey was conducted among amateur bodybuilders on their perceptions of the consumption of dietary supplements [16]. The research identified three main reasons for taking supplements: to achieve an ideal body shape, to increase strength/muscular power, and to enhance performance, and it found that the benefits of consumption outweigh the potential negative consequences. Body image, as represented by sport and Western fitness culture, can trigger both positive and negative behaviors related to supplement consumption. Amateur bodybuilders feel the need to take supplements to achieve the desired body image or to perform successfully in a competitive environment. In addition, another motivational factor could be reducing fatigue [17,18].

There is a certain gender polarization in the motivations for dietary supplement intake: while female respondents consume supplements for health maintenance or in the context of a diet with a focus on fat loss [8], men focus on increasing speed and explosiveness and gaining weight and/or muscle mass. Socio-cultural pressures on men have led many of them to seek to assert their social and gender identity through hypermasculinity [19]. As a result, male bodybuilders, for example, typically use dietary supplements and various weight-gain agents with high frequency [20]. The percentage of regular dietary supplement consumers was high among men exercising at commercial gyms in New York (84.7%) [21], and among men exercising at gyms in a city in Brazil (82.8%) [22], and the reported supplement use in this study was in line with the prevalence reported for male Swedish fitness customers (94%) [23]. A review by Hulteen et al. [24] aimed to determine the most popular physical activities performed by children, adolescents, and adults globally. Statistical bureau websites and article databases of Scopus, ProQuest, SPORTDiscus, and Science Direct were searched. According to the study’s findings, the child and adolescent participation results were highly dependent upon region. Global data for adults show a consistent pattern of running and walking involvement.

### 1.2. The Relationship between Dietary Supplement Consumption and Sport Performance

Not only do dietary supplement consumption patterns differ by motivation, but also by exercise intensity. Of course, proper nutrition can have a significant impact on sports performance. However, physical activity itself has several benefits, including an increase in lean body mass and an increase in the resting metabolic rate, which have a positive effect on appetite. Furthermore, the pressure to achieve a greater sporting performance or an ideal body image is a strong motivation for young athletes. Kiss and co-authors [25] found that the consumption of dietary supplements among recreational sports participants is directly related to the intensity of the sporting activity and the perceived importance of their consumption.

Studies among bodybuilders [26] show that a wide range of supplements are used in this sport: protein powder, BCAAs (branched-chain amino acids), vitamin C, omega 3 fatty acids, multivitamins, creatine, vitamin D, pre-workout supplements, carbohydrate complexes, amino acids, fat burners, minerals, joint supplements, protein slices, and other supplements. Among male leisure-time fitness athletes, it was found that the top 10 supplements used by younger men, in descending order, were as follows: protein powders, energy drinks, multi-vitamins, creatine, vitamin C, glutamine, energy bars, protein bars, amino acids, and recovery drinks [27]. There has also been an increase in the consumption of protein supplements among recreational athletes [13]. The typical dietary supplement intake of amateur tennis players consists of sports drinks, energy bars, vitamin complexes, proteins, and creatine, according to a study [28]. In terms of differences in the consumption of dietary supplements, the results of Sánchez-Oliver et al. [29] suggest that respondents typically consume various products (protein, caffeine, sports drinks, energy bars, creatine monohydrates) to enhance their sports performance, and amateur athletes tend to consume supplements more intensively in connection with competitions. The responses also showed that the amateur population focuses on pre-workout intake. Incidentally, although it is not the subject of the present study, belonging to a particular nutritional segment (e.g., vegan) also coincides with significant specificities in the intake of supplements [30].

In a survey of amateur female runners conducted by Locquet et al. [31], 34.6% reported that they had consumed a self-administered pill and supplement “package” before a race to enhance their running performance. The research also shows that the consumption of various medicines and supplements increases in proportion to the intensity of the sport activity. Respondents reported that they perceived a positive impact on their physical fitness and sports performance by taking dietary supplements [28]. This serves as a kind of motivation for consumers, as the use of dietary supplements promises to result in an improved sports performance. It is necessary to mention herein the so-called multi-ingredient pre-workout dietary supplements (MIPSs), which are showing a growing trend in their significance [32,33]; however, the current research does not address this.

This study adds to the current academic literature in the following ways. First, it describes the dietary supplement usage in the segment examined. Second, it explores the driver factors of dietary supplementation. Third, the present study was set up to investigate the motivational segments.

## 2. Materials and Methods

### 2.1. Study Design and Participants

In this study, in a quantitative approach, telephone interviews were conducted. The choice of telephone interviews was justified by the fact that this would significantly reduce the schematic response rate typical of self-administered questionnaires. Response cooperation was rewarded via an incentive of randomly distributed shopping coupons. The survey was conducted in the first quarter of 2021 with random-dialing sampling. The thereby attained population was pre-filtered: respondents with a minimum of 3 sports activities a month were interviewed. In addition, the demographic composition was balanced in terms of gender, while age groups were unevenly distributed, with the first three age groups making up 30%, and the oldest one making up around 10%. The data collection resulted in a total of 236 valid responses, while 17% of the gross sample was excluded due to refused or uncompleted responses.

As per the preliminary criteria, the gender distribution of respondents was nearly even (45.0% male; 55.0% female). By age group, however, the sample structure was intentionally asymmetrically weighted, reducing the number of respondents in the 45+ age segment. Thus, the age composition of the data analyzed was as follows: 18–24 years old: 30.0%; 25–34 years old: 30.0%; 35–44 years old: 29.0%; and 45–54 years old: 11.0%. Almost two-thirds of respondents participated in sports several times a week (60.4%), with the second most common response being once a day (17.9%). The sample was less extreme, with 6.4% of respondents participating in sports daily, 8.5% once a week, and 6.8% from 1 to 3 times a month. According to the preliminary filter, those who played sports a minimum of 3 times per month were included in the analysis.

A total of 81% of respondents participated in leisure-time sports; thus, the results provide more in-depth information on the consumption and motivations for leisure-time sports-related supplement consumption. In terms of sport type, aerobic sports predominated. Running (42.5%) was the most frequently mentioned by respondents, while weight training (gym) and bodybuilding were also mentioned (37.1%). Fitness (34.9%) was the third most common leisure-time sport, followed by cycling (28.0%), aerobics (14.5%), and yoga (14.5%). Leisure-time sports were moderately dominated by team sports, with football being the most frequently mentioned (7.0%), followed by basketball (4.3%), volleyball (3.2%), and handball (1.6%).

Respondents’ participation in the study was entirely consensual, anonymous, and voluntary.

### 2.2. Questionnaire Design and Analysis

To achieve the research objective, a structured survey was conducted. The survey consisted of several research themes. The questionnaire started with demographic data (gender, age), then asked about the regularity (several times a day; 1× per day; several times a week; once a week; 3 times a month) and the framework of the sport activity (leisure activities; association-related (competitive) sport). The questionnaire also asked about the type of sport activity, where several sports could be mentioned (29 multiple-choice answers and an “optional” answer). Eleven attitudinal statements related to sport motivations were then included. Thereafter, the questionnaire focused on the topic of dietary supplements (vitamins included). The first question was whether the respondent consumed any supplements, and if so, what types, how often, and for what purpose. The measurement scales were categorical (nominal) or ordinal ones. Regarding the consumption, the respondents had to choose between “Yes, I consume.”; “I don’t consume it, but I plan to in the future.”; “I don’t consume and I don’t plan to.”. Thus, the research also took into account the individuals that planned to engage in some form of dietary supplement consumption. As for the types of nutritional supplements, multiple answers could be chosen among the following: vitamins, minerals; amino acids, proteins; essential fats (e.g., omega-3, omega-6); other bodybuilding supplements (combined protein drinks, BCAAs, glutamine, creatine, testosterone boosters); and an “other” option as well. We were interested in the frequency of the consumption of each dietary supplement among the respondents. Their task was to provide the consumption frequency for each dietary supplement category separately as follows: many times a day/1× per day/several times a week/1× a week/a few times a month/never. As for the purpose of consuming nutritional supplements, the options were “Preserving health”; “Increasing muscle mass”; “Cartilage protection”; “Performance enhancement”; “Improving physical well-being”; and an “Other” option.

The data-cleaning procedure included checking for missing data and respondent bias. To detect and exclude extreme values, a case anomaly index was used. In the final database of 236 respondents, the proportion of missing data for each survey question was less than five percent.

The descriptive statistical analyses were conducted using SPSS Statistics 28.0. Sample characteristics were analyzed applying descriptive statistics to the data. Analyzing the data descriptive statistical methods, descriptive statistical indicators and cross-tabulation analyses were used. The statistical-significance approach was used at a 5% significance level. To measure the relative (strength) of an association for nominal variables, Cramer’s V measure was used. Association correlation tests were used to examine significant differences between segments. Taking the factors of the motivations as a basis, K-means clustering and the Euclidean distance method were applied.

### 2.3. Limitations

The research was limited by demographic, age-group, and geographical constraints. The results of the present study should be interpreted in light of the fact that the sample was comparable to but not representative of the Hungarian population, which means that the results are not fully generalizable. The second is a demographical limitation, as the research considered only the age group of 18–54. However, it should be underlined that the age disproportion of the sample was planned and improved the results, as the age group that is active in leisure sports was studied. It is important to note that only marketed dietary supplements were analyzed in this research. Steroids, hormone-replacement therapies, doping drugs, and all other illegal substances were excluded.

## 3. Results

### 3.1. Dietary Supplement Consumption Patterns

Within the 236-item sample, the proportion of active dietary supplement consumers was 83.0% (191 people). A total of 11.0% of the respondents did not currently consume dietary supplements but planned to do so in the future, and only 6% neither consumed nor planned to consume them. The analysis, therefore, was conducted on the subsample of 191 individuals of the total sample of 236 items.

The socio-demographic characteristics of the respondents who participated in leisure-time sports and, in addition, used dietary supplements (191 people) showed a slight shift towards female leisure-time athletes, with 43.5% male and 56.5% female respondents. By age group, the 18–24 one made up 33.0%, the 25–34 one 25.0%, the 35–44 one 31.0%, and the 45–54 group 11.0%. In terms of the frequency of exercise, sporting activity several times a week (66.5%) was also the most common response among those taking supplements, with daily sport being the second most common response at 13.1%. Of the respondents who did not take a dietary supplement but planned to in the future, 75.0% were younger men aged 18–24 who exercised several times a week.

When looking at the categories of the consumption of food supplements, clearly most people (95.0%) consumed vitamins and minerals from “external” sources. The amino acid and protein preparations were the second most often consumed dietary supplement (59.5%), followed by essential fats (40.0%), and finally, other bodybuilding supplements (37.0%). As for bodybuilding supplements, they are products designed to support and enhance the physical performance, muscle growth, and overall athletic goals of individuals engaged in bodybuilding and other resistance training activities. It is interesting to note the differences in the frequency of the consumption of different types of dietary supplements by age group. The results obtained show that the consumption of vitamins and minerals was typical for all age groups, while the response “several times a day” appeared for those aged 35 and over (the age groups 35–44 (25.8%), 45–54 (34.8%), and 25–34 (31.9%) consumed dietary supplements several times a day). For amino acids and proteins, there was no age-specific pattern, while the consumption of essential fatty acids was more typical of those aged 25 and over and increased with age. The consumption of other bodybuilding supplements once a day or several times a week was most common among the 25–34 age group. In the other answer category, a total of five respondents marked it, and of the responses, two answers fell into the given categories and three mentioned all three fat-burning categories.

When looking at the sporting characteristics of active dietary supplement users, the highest proportion of participation in aerobic and cardio equipment-free sports (65.3%) was found. Aerobic equipment-intensive sports (11.4%) also featured strongly, and a high proportion participated in fitness sports (19.9%). The different categories were named based on whether athletes used any equipment for recreational sports or engaged in them without using any equipment. Team sports were less prevalent in the leisure-time sport activities of the sample surveyed (3.4%), appearing less markedly in the 35–44 and 45–54 age groups, while in the 18–24 and 25–34 age cohorts, these were performed together with aerobic and cardio equipment-free sports.

Most of the sample (62.7%) participated in sports several times a week, 17.9% participated daily, and 7.5% participated more than once a day. Among those who participated in sports less frequently, 8.0% of the respondents participated weekly, and 4.0% participated 1–3 times a month. Less frequent exercisers were significantly more likely (*p* = 0.002, Cramer’s V = 0.298) to engage in aerobic equipment sports (37.5%), while aerobic equipment-free sports were typically mentioned at higher frequencies. The proportion participating in fitness sports was higher among those who exercised daily or more than once a day (47.0%), while it ranged from 12.5% to 17.5% for the other exercise frequencies (*p* = 0.002, Cramer’s V = 0.298).

Overall, there was a high prevalence of vitamin supplement consumption among people who participated in leisure-time sports and actively took dietary supplements. There was a significant variation in the sample in terms of the type of sport and frequency of participation, and it is noticeable that in the younger age group, different team sports appear alongside aerobic equipment-free sports activity.

### 3.2. Relationship between Leisure-Time-Sport Type and Dietary Supplement Consumption

It is also interesting to look at the frequency of the food supplement consumption associated with each sport category (see Table 1). In this regard, the consumption frequency of vitamins for aerobic/cardio and fitness sports tended to be typically several times a day or once a day. The consumption of amino acids and proteins was dominated by sports in the fitness category, and participants typically consumed several times a day, once a day, or several times a week. This group of athletes was also characterized by a once-daily intake of essential fats, and this supplement was also a preferred supplement for those who engaged in equipment-free aerobic/cardio exercise. It should be noted that there was a certain number of “never” answers regarding fats, presumably for two reasons: it is possible that leisure-time sports athletes are not aware of the importance of consuming essential fats, and there may be a preconception that “fat is fattening”, and thus a fear of directly consuming macronutrients. The consumption of other bodybuilding supplements was reported for strength/fitness sports (28.0%) and aerobic/cardio exercise (25.0%) on a daily or weekly basis.

### 3.3. Motivational Drivers for the Consumption of Dietary Supplements

**Figure 1:**
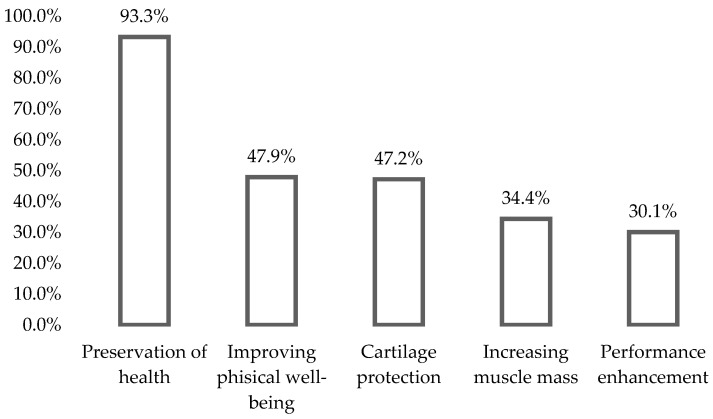
Motivational factors of dietary supplement consumption among leisure-time athletes actively consuming dietary supplements.

The main motivator for taking supplements was the preservation of health, followed by improving physical well-being, protecting cartilage, increasing muscle mass, and finally, enhancing performance (see Figure 1).

When the purposes of supplement consumption are analyzed by sport category, health maintenance and improvement in physical well-being were predominant for all groups. However, in the case of aerobic/cardio sports that do not require the use of equipment (e.g., running on asphalt), cartilage protection was predominant (51.2%), while for those who participated in fitness, cartilage protection and performance enhancement (42.1%) were also primary objectives. For non-instrumental aerobic sports (e.g., running), mainly women (62%) typically exercised daily (20%) or several times a week (68%) (by age: 18–24: 33%; 25–34: 25%; 35–44: 31%; 45–54: 11%). For equipment-intensive aerobic sports (e.g., cycling), more men can be identified (60.0%) who typically exercised several times a week (45.0%), on one–three occasions a month (30.0%), once a week (20.0%), or daily (only 5.0%). By age, they tended to be older within the sample, with 35% in the 35–44 age group and 20.0% in the 45–54 age group. In terms of the distribution of fitness athletes within the sample, 92.0% were under 44 years old, and 80.0% exercised more than once a week, with a roughly 50/50 gender split between men (52%) and women (48%).

When looking at the purpose of consuming dietary supplements by age group, it can be said that the preservation of health as a motivation cuts across age categories, while the protection of cartilage and improvement in physical well-being are more prevalent in the 25+ and 35+ age groups, and the consumption of supplements to increase muscle mass and enhance performance is skewed towards the younger end of the sample. While increasing muscle mass as a motivating force is more predominant in the 18–34 age group (*p* = 0.042, Cramer’s V = 0.121), cartilage protection is significantly higher in the older age group over 25 (*p* = 0.000, Cramer’s V = 0.341). There is no significant difference between age groups for improving physical well-being (see Table 2).

### 3.4. Segment Structure Based on Consumption Motivational Drivers

Examining the segment structure of consumers via cluster analysis, three clusters could be obtained. The cluster denominations are as follows: *Young performance-freaks* (Cluster 1), *Health-conscious mass gainers* (Cluster 2), and *Empowered health queens* (Cluster 3). These groups proved to be significantly characteristic in the following dimensions:

- In terms of gender, there is a shift towards the women’s segment from Cluster 1 through to Cluster 3 (Cluster 1: 50% male and 50% female; Cluster 2: 47% male and 53% female; Cluster 3: 36% male and 64% female);
- The centroid of age increases from Cluster 1 through to Cluster 3;
- The frequency of sports activity increases across clusters.

The motivation profile of the segments can be seen in Table 3.

Respondents who took dietary supplements solely for health-promoting purposes were mostly women (66.7%), of whom 18.5% were aged 18–24, 29.6% were aged 25–34, 48.1% were aged 35–44, and 3.7% were aged 45–54. In terms of the frequency of their sporting activity, most played sports several times a week (51.9%) or once a week (29.6%). A further 11.1% exercised less frequently (1–3 times a month), as well as consumed various dietary supplement products for health promotion purposes. Where sports categories are concerned, 70% of them played equipment-free aerobic/cardio sports and 14.5% played aerobic/cardio equipment sports. All of them regularly consumed vitamins and minerals: amino acids and proteins: 11.1%; essential fats: 25.9%; and other bodybuilding supplements: only 7.4%.

Where vitamins and minerals are concerned, responses indicating that these were taken several times a day (15%) or once a day (74%) were the most typical, while 3.7% never took them. The sample took amino acids and proteins less often: once a day (7.4%) or several times a week (3.7%), and 88.9% did not consume them at all. Essential fats were consumed once a day (11.1%) or several times a week (7.4%), while 25.9% consumed them once a week. A total of 74.1% stated that they never consumed essential fats. This group typically never took other bodybuilding supplements, with only 7.4% of them consuming them several times a week.

A majority of the respondents who took supplements only for health reasons were women (66.7%), with men comprising 33.3% of this group. In terms of all the respondents’ ages, 18.5% were aged 18–24, 29.6% were aged 25–34, 48.1% were aged 35–44, and 3.7% were aged 45–54.

**Figure 2:**
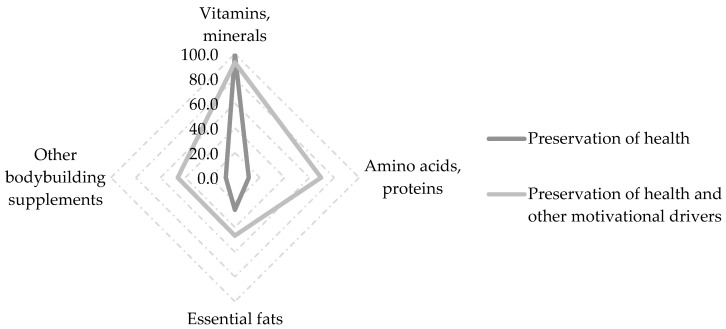
Comparison: the group that mentioned only health maintenance and other motivations.

It appears that the preservation of health as a motivational driver was the foremost consideration for all three leisure-time-athlete segments. Examining the consumption motivations in more detail, there appears to be respondents who consumed dietary supplements exclusively for health preservation purposes, mainly vitamins and minerals, in addition to essential fats. The use of amino acids and proteins or other bodybuilding supplements seems to make up only a low proportion of this group (Figure 2).

## 4. Discussion

The aim of this study was to investigate the consumption habits related to diet supplementation, as well as the motivational drivers behind using dietary supplements, among leisure-time athletes. The scientific relevance of studying the consumption habits related to diet supplementation and the motivational drivers behind using dietary supplements among leisure-time athletes is vast and can be underlined as follows:

- Understanding nutritional requirements: Leisure-time athletes often have nutritional needs that differ from those of sedentary individuals or elite athletes. Researching their consumption habits can provide insights into whether they are meeting these needs and how supplements contribute to their overall nutritional intake;
- Health implications: Dietary supplements can both benefit and harm health. Certain supplements may enhance performance, recovery, or general health, while others may lead to adverse events if misused or overconsumed;
- Understanding consumption habits could help mitigate health risks and maximize benefits: overall, this research contributes to the broader field of public health by enhancing our understanding of dietary behaviors, health outcomes, and risk factors among a significant population group;
- Psychological factors: Investigating the motivational drivers behind supplement use can reveal psychological factors, such as the desire for improved performance, body image concerns, or the influence of peer or societal pressures. This can help inform interventions to promote healthier attitudes and behaviors around supplement use;
- Economic factors: Dietary supplements represent a significant economic market. Understanding consumer behavior in this context can inform strategies for product development, marketing, and policy making;
- Policy and regulation: Studying consumption habits and motivations can inform regulatory approaches and policy making in relation to the supplement industry. This could lead to the improved safety, efficacy, and quality control of dietary supplements;
- Education and awareness: studies in this field can help identify gaps in knowledge and misconceptions about dietary supplements among leisure-time athletes, which can then be addressed through targeted education and awareness campaigns;
- Personalized nutrition: the study can contribute to the growing field of personalized nutrition by examining how individual factors (e.g., gender, age, fitness level, genetic makeup) influence supplement use and response.

Related to the above, the increasing importance of area is seen also, for example, in the policy of the *Nutrients* journal, which is going to launch numerous Special Issues on the topic of dietary supplement consumption in 2023–2024.

The results of this study show patterns of dietary supplement consumption among those aged 18–54. Consumption patterns for vitamin supplements, essential fats, amino acids, proteins, and other bodybuilding supplements show differences that reflect the type of sport involved. It should be mentioned that attitude-based consumption—due to the relative stability of consumer attitudes—has presumably remained unchanged in the 2 years since data collection.

Most amateur athletes surveyed in the study were considered potentially stable users of dietary supplements. Only 10% of them did not take or plan to take supplements. The levels of vitamin supplementation were particularly high in the age groups studied (95.0%), and amino acid/protein supplementation was present in 60.0% of the participants. Daily intake or multiple daily intakes of vitamin supplements was the case for almost 80.0% of the participants, while daily intake or multiple daily intakes of amino acids, proteins, and essential fats was also true for one-quarter of the respondents.

In terms of the frequency of exercise, the survey participants participated in sport activities several times a week (51.9%) or once a week (29.6%). It can also be stated that the proportion of those who exercised less frequently and consumed various food supplements for health purposes was 11.1%. Where the category of sport is concerned, 70.0% of them participated in equipment-free aerobic and cardio sports, and 14.5% participated in aerobic and cardio equipment-intensive sports. Of these, all respondents regularly consumed vitamins and minerals, 11.1% consumed amino acids and proteins, 25.9% consumed essential fats, and only 7.4% consumed other bodybuilding supplements. 

In terms of the consumption of vitamins and minerals, several times a day or once a day (altogether 77.2%) were the most common frequencies of consumption. However, 3.7% of the respondents never consumed products in this supplement category. Amino acids and proteins were consumed less frequently: daily (7.4%) or several times a week (3.7%). A total of 88.9% of the examined segment did not consume them at all.

Finally, essential fats were consumed once a day (11.1%), several times a week (7.4%), or once a week (25.9%). A total of 74.1% stated that they never consumed essential fats. Other bodybuilding supplements were typically never consumed, with only 7.4% consuming more than several times a week.

The three groups showed a significant difference in the dietary supplement intake for each dietary supplement category (*p* = 0.0023; Cramer’s V = 0.234), with a significant difference for aerobic and cardio equipment-free and aerobic and cardio equipment-intensive sports. A significant finding is that aerobic exercise had a particularly high proportion of respondents (almost half of them) who never consumed amino acids, proteins, or essential fats (aerobic equipment-free: 58%; and aerobic equipment-intensive: 71%).

Patterns in the consumption of dietary supplements differed for each type of sport, with the only difference being the high consumption of vitamin supplements. For aerobic and cardio equipment-free (91%), aerobic and cardio equipment-intensive (88%), and fitness (85%) sports, of those consuming vitamin supplements, they did so frequently: several times a day, once a day, or several times a week.

Based on the results obtained, we found that higher levels of supplement consumption than the rates reported in the literature [26] were mainly observed in the consumption of vitamin preparations. The finding of Hartmann and Siegrist [13], that protein consumption is a prominent component of dietary supplement consumption, could not be confirmed.

Meanwhile, individual sport practice, rather than team sports, was associated with a higher likelihood of food supplement use. In line with earlier research results [34], multivitamins and minerals were commonly used. The study of Mazzilli et al. [17] aimed at evaluating the prevalence of the use of dietary supplements (DSs) among gym users and gym instructors involved in body-shaping-oriented fitness training. The results showed a high prevalence in the use of DSs in the population considered, with 85.4% of the participants declaring they used DSs, with high heterogeneity in the numbers and combinations used. Hamilton et al. [35] also found significant differences among divisions in race, ethnicity, sport dietitian access, name, image, and likeness (NIL), advice to consume DSs, and knowledge of DSs.

The results show that the consumption of these products is mainly driven by positive health preconceptions. Strikingly, performance enhancement and increasing muscle mass were statistically less considered as the main reasons for taking food supplements. Instead, they were used to enhance physical well-being or cartilage protection.

Although the results are based on a relatively small number of selected participants (n = 235), the research design applied a thorough examination of the motivational drivers and sport-type-related consumption patterns. Overall, the findings show that dietary supplementation is linked to health preservation and physical well-being in a segment of leisure-time athletes.

## 5. Conclusions and Implications

### 5.1. Conclusions

Within the field of research on sport-related patterns of dietary supplement consumption among recreational athletes, there are only a few research results available in the international literature. Hence, the results of this study—filling a research gap—can be considered novel.

The results were obtained from this sample size; of course, with a larger sample size, a smaller bias could be expected. Another limitation is that the survey was conducted in the Hungarian market, and so it is valid only within this geographical area.

In sum, the study highlights the motivational drivers and patterns of dietary supplement consumption among leisure-time athletes. The findings shed light on the varying motivations based on sport type and age, emphasizing the importance of informed supplement use for health preservation, while being cautious about the potential risks associated with excessive intake. Consumers who consume only for health purposes are significantly different: they typically do not consume any other food supplements, only vitamins and minerals. Further research with larger and more diverse samples could deepen our understanding of supplement consumption in these contexts.

### 5.2. Implications

Societal: From a societal perspective, overconsumption can also be harmful to health and is to be avoided from a sustainability point of view. Consumers’ knowledge needs to be monitored continuously. This calls for both knowledge assessment and the dissemination of information;

Academic: The academic implication is that the survey opens an opportunity for further research, for example, to study other generations or to conduct international comparative analyses on dietary supplement usage. Other future research directions may include investigations into “health purposes”, consumers’ vitamin and mineral intake habits, or the excessive intake of dietary supplements from the perspective of the psychology of consumption;

For practice: So far as managerial implications are concerned, it should be noted that, based on the appearance of different motivations in the segments examined, this study justifies assigning a differentiated communication focus to each leisure-time-athlete segment. Companies involved in the production and marketing of dietary supplements should embrace socially responsible communication. This includes clear labeling, transparent information regarding the benefits and potential risks, and promoting conscious consumption patterns.

## Figures and Tables

**Table 1:** Comparison of the consumption of dietary supplement categories in different sport types.

	Consumption Frequency	Equipment-Free Aerobic/Cardio	Aerobic/Cardio Equipment Sports	Team Sports *	Fitness Sports	Total
Vitamins, minerals	Several times a day	18.5%	5.9%	25.0%	35.0%	19.3%
	1× a day	61.5%	70.6%	25.0%	30.0%	57.9%
	Several times a week	11.5%	11.8%	25.0%	20.0%	12.9%
	1× per week	0.0%	0.0%	0.0%	5.0%	0.6%
	Several times a month	0.8%	0.0%	0.0%	0.0%	0.6%
	Never	7.7%	11.8%	25.0%	10.0%	8.8%
Amino acids, proteins	Several times a day	6.2%	0.0%	0.0%	10.0%	5.8%
	1× a day	21.5%	11.8%	0.0%	25.0%	20.5%
	Several times a week	21.5%	29.4%	0.0%	30.0%	22.8%
	1× per week	4.6%	11.8%	0.0%	0.0%	4.7%
	Several times a month	2.3%	0.0%	0.0%	5.0%	2.3%
	Never	43.8%	47.1%	100.0%	30.0%	43.9%
Essential fats	Several times a day	2.3%	5.9%	0.0%	5.0%	2.9%
	1× a day	26.2%	11.8%	0.0%	25.0%	24.0%
	Several times a week	6.9%	5.9%	0.0%	5.0%	6.4%
	1× per week	4.6%	0.0%	0.0%	0.0%	3.5%
	Several times a month	1.5%	5.9%	0.0%	0.0%	1.8%
	Never	58.5%	70.6%	100.0%	65.0%	61.4%
Other bodybuilding supplements	Several times a day	5.4%	0.0%	0.0%	10.0%	5.3%
	1× a day	10.0%	0.0%	0.0%	10.0%	8.8%
	Several times a week	18.5%	11.8%	0.0%	15.0%	17.0%
	1× per week	3.8%	5.9%	0.0%	0.0%	3.5%
	Several times a month	0.8%	0.0%	0.0%	0.0%	0.6%
	Never	61.5%	82.4%	100.0%	65.0%	64.9%

* Note: the number of respondents playing team sports is low.

**Table 2:** Comparison of the motivational factors for consuming dietary supplements by age group.

Age Group	Preservation of Health	Increasing Muscle Mass	Cartilage Protection	Performance Enhancement	Improving Physical Well-Being
18–24	88.7%	43.4%	34.0%	37.7%	37.7%
25–34	95.7%	42.0%	60.9%	49.3%	47.8%
35–44	96.7%	31.1%	50.8%	29.5%	59.0%
45–54	86.4%	31.8%	68.2%	22.7%	45.5%

**Table 3:** Comparison of the compositions and motivation factors of the segments.

		Young Performance-Freaks 19.7%	Health-Conscious Mass Gainers 35.7%	Empowered Health Queens 44.6%
Gender	Male	50.0%	47.4%	35.8%
	Female	50.0%	52.6%	64.2%
Age group	18–24	**38.1%**	28.9%	22.1%
	25–34	**31.0%**	**38.2%**	28.4%
	35–44	23.8%	25.0%	**34.7%**
	45–54	7.1%	7.9%	14.7%
Sports frequency	Several times a day	14.3%	10.5%	1.1%
	1× a day	**21.4%**	**21.1%**	11.6%
	Several times a week	**57.1%**	**64.5%**	**63.2%**
	1× per week	2.4%	2.6%	**15.8%**
	Monthly 1–3 occasions	4.8%	1.3%	8.4%
Vitamins, minerals	Several times a day	**23.8%**	25.0%	20.0%
	1× a day	**33.3%**	**53.9%**	**67.4%**
	Several times a week	16.7%	11.8%	10.5%
	1× per week	0.0%	1.3%	0.0%
	Several times a month	2.4%	1.3%	0.0%
	Never	23.8%	6.6%	2.1%
Amino acids, proteins	Several times a day	4.8%	25.0%	0.0%
	1× a day	28.6%	28.9%	16.8%
	Several times a week	28.6%	30.3%	9.5%
	1× per week	2.4%	0.0%	7.4%
	A few times a month	2.4%	0.0%	4.2%
	Never	33.3%	15.8%	62.1%
Essential fats	Several times a day	0.0%	7.9%	3.2%
	1× a day	**28.6%**	**30.3%**	**22.1%**
	Several times a week	4.8%	11.8%	8.4%
	1× per week	0.0%	1.3%	5.3%
	A few times a month	0.0%	1.3%	2.1%
	Never	66.7%	47.4%	58.9%
Other bodybuilding supplements	Several times a day	4.8%	17.1%	1.1%
	1× a day	**19.0%**	**26.3%**	0.0%
	Several times a week	**16.7%**	**30.3%**	5.3%
	1× per week	2.4%	2.6%	3.2%
	A few times a month	2.4%	0.0%	0.0%
	Never	54.8%	23.7%	**90.5%**
Motivation	Preserving health	**59.5%**	**94.7%**	**98.9%**
	Protecting cartilage	31.0%	**63.2%**	47.4%
	Increasing muscle mass	4.8%	**100.0%**	0.0%
	Boosting performance	**81.0%**	**56.6%**	0.0%
	Improving physical well-being	40.5%	47.4%	48.4%
Sport category	Aerobic and cardio equipment-free	59.5%	61.8%	**74.7%**
	Aerobic and cardio equipment required	9.5%	10.5%	11.6%
	Team sports	2.4%	0.0%	5.3%
	Fitness	**28.6%**	**27.6%**	

## Data Availability

The dataset is available in the Hungarian language and is available from the authors upon reasonable request.
